# Vitamin D_3_ inhibits p38 MAPK and senescence‐associated inflammatory mediator secretion by senescent fibroblasts that impacts immune responses during ageing

**DOI:** 10.1111/acel.14093

**Published:** 2024-01-29

**Authors:** Souraya Sayegh, Carlos Henrique Fantecelle, Phatthamon Laphanuwat, Priya Subramanian, Malcom H. A. Rustin, Daniel C. O. Gomes, Arne N. Akbar, Emma S. Chambers

**Affiliations:** ^1^ Division of Medicine University College London London UK; ^2^ Núcleo de Doenças Infecciosas Universidade Federal do Espírito Santo Vitoria Brazil; ^3^ Department of Dermatology Royal Free Hospital London UK; ^4^ Centre for Immunobiology, Blizard Institute Queen Mary University of London London UK

**Keywords:** ageing, p38 MAPK, SASP, senescence, skin, vitamin D

## Abstract

Vitamin D_3_ replacement in older insufficient adults significantly improves their antigen‐specific varicella zoster virus (VZV) cutaneous immunity. However, the mechanisms involved in this enhancement of cutaneous immunity are not known. Here, we show for the first time that vitamin D_3_ blocks the senescence‐associated secretory phenotype (SASP) production by senescent fibroblasts by partially inhibiting the p38 MAPK pathway. Furthermore, transcriptomic analysis of skin biopsies from older subjects after vitamin D_3_ supplementation shows that vitamin D_3_ inhibits the same inflammatory pathways in response to saline as the specific p38 inhibitor, losmapimod, which also enhances immunity in the skin of older subjects. Vitamin D_3_ supplementation therefore may enhance immunity during ageing in part by blocking p38 MAPK signalling and in turn inhibit SASP production from senescent cells in vivo.

## Graphical abstract

Ageing is associated with decreased cutaneous immunity and increased inflammageing. Senescent dermal fibroblasts increase in the skin and secrete cytokines and chemokines known as the senescence‐associated secretory phenotype (SASP) that is regulated in part by p38 MAP kinase. Here we show for the first time that vitamin D_3_ inhibits the p38 MAPK pathway in senescent fibroblasts to block the SASP. This reduces the inflammatory environment in the skin leading to increased cutaneous immunity during ageing.

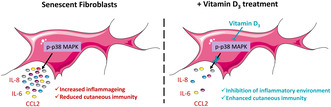

**Abbreviations**
CRP: C‐reactive protein
DEGs: differentially expressed genes
MKP‐1: MAPK phosphatase‐1
p38 MAPK: p38 MAP kinase
PGE2: prostaglandin E2
RNA‐Seq: RNA‐sequencing
SASP: senescence‐associated secretory phenotype
SVs: surrogate variables
TRM: resident memory T cells
VDR: vitamin D receptor
VZV: varicella zoster virus

## 1 INTRODUCTION, RESULTS AND DISCUSSION

Ageing is associated with increased frailty and reduced immunity to many pathogens, highlighted by the re‐activation of latent infections and reduced vaccine efficiency (Ciabattini et al., 2018; Diffey & Langtry, 2005; Furman et al., 2017; Gavazzi & Krause, 2002). This decline in immune reactivity is associated with chronic low‐grade systemic inflammation termed ‘inflammageing’, that is characterized by elevated serum concentrations of IL‐6, TNF‐α and C‐reactive protein (CRP) (Franceschi et al., 2017). Furthermore, increased inflammageing is correlated with a sub‐optimal vitamin D_3_ status (Ju et al., 2018; Laird et al., 2014). We showed recently that older humans who have low serum 25‐hydroxyvitamin D_3_ exhibit increased non‐specific inflammatory responses in the skin after cutaneous challenge with saline (sterile inflammation) (Chambers, Vukmanovic‐Stejic, Turner, et al., 2021). This increased propensity to mount non‐specific inflammation in the skin is significantly correlated with decreased ability of the same individuals to respond to challenge with recall antigens such as varicella zoster virus (VZV) in the skin (Chambers, Vukmanovic‐Stejic, Turner, et al., 2021). This demonstrates an association between low levels of vitamin D_3_, non‐specific inflammation (inflammageing) and decreased immunity in individuals during ageing.

The non‐specific cutaneous inflammation observed in older adults was driven by recruitment of inflammatory monocytes from the circulation by CCL2 secretion from senescent fibroblasts (Chambers, Vukmanovic‐Stejic, Shih, et al., 2021). Senescent dermal fibroblasts increase in the skin during ageing and secrete an array of cytokines and chemokines known as the senescence‐associated secretory phenotype (SASP), that is regulated in part by p38 MAP kinase (p38 MAPK) (Pereira et al., 2019). CCL2, the chemoattractant for CCR2^+^ inflammatory monocytes, is a known component of the SASP (Chambers, Vukmanovic‐Stejic, Shih, et al., 2021). Once recruited, these inflammatory monocytes inhibited the proliferation and function of resident memory T cells (TRM) through the production of prostaglandin E2 (PGE2). This leads to the decreased cutaneous recall response after VZV challenge (Chambers, Vukmanovic‐Stejic, Shih, et al., 2021).

A key observation was that vitamin D_3_ replacement for 14 weeks in older adults significantly enhances their VZV‐specific cutaneous immunity. We now show that vitamin D_3_ significantly inhibits the SASP via p38 MAPK in senescent fibroblasts. Transcriptomics analysis of skin biopsies after vitamin D_3_ supplementation show that vitamin D_3_ reduces inflammatory monocytes infiltration in vivo similarly to losmapimod, a specific p38 inhibitor. This provides a mechanism for the enhancement of the recall response to VZV challenge in older adults.

**FIGURE 1:**
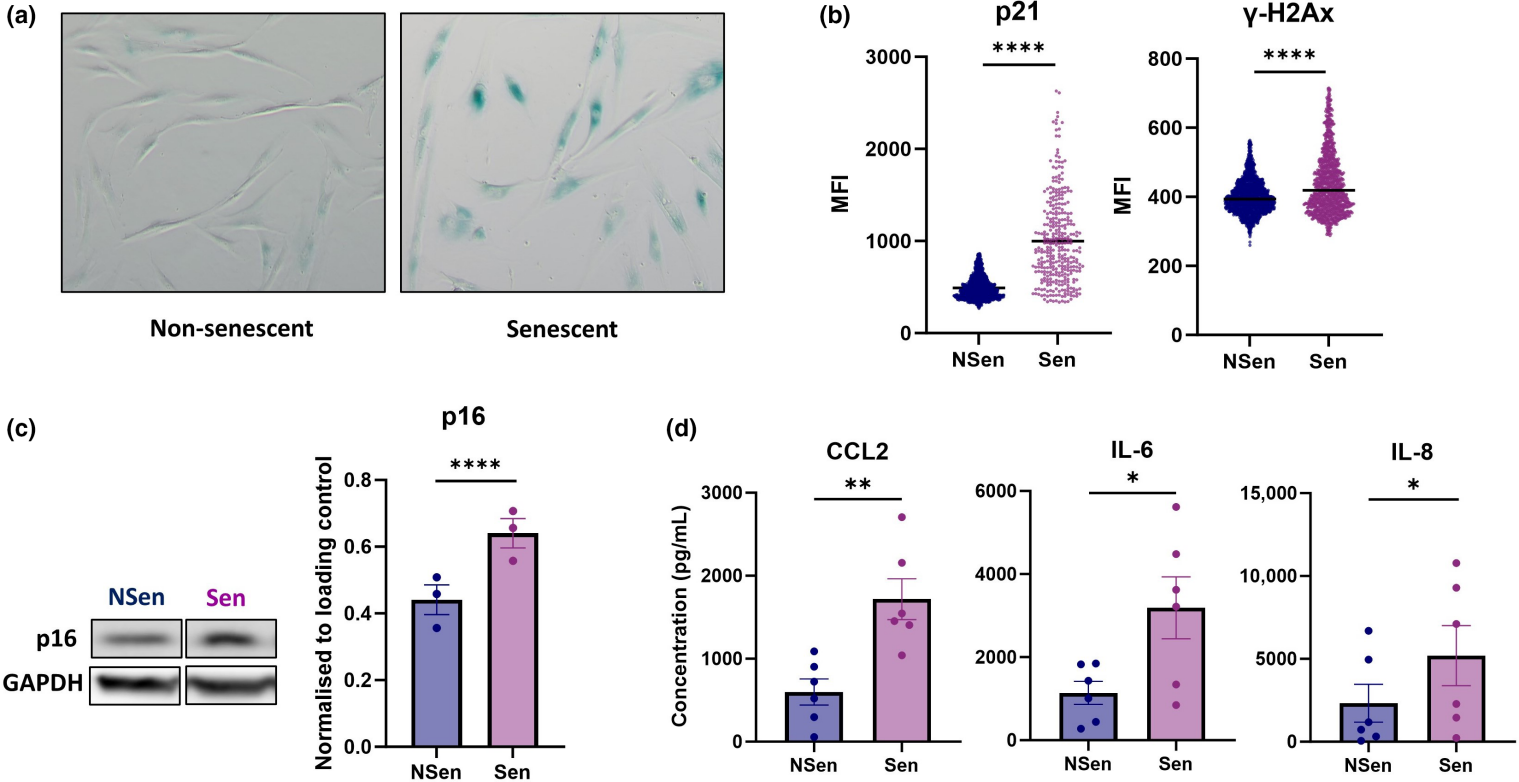
Induction of senescent fibroblasts in vitro. Human dermal fibroblasts were isolated from skin biopsies, and senescence was induced using 10 Gy x‐ray ionising radiation. (a) Senescence was evaluated using an SA‐β‐galactosidase assay. (b) Expression of p21 and γ‐H2Ax was evaluated using immunofluorescence (Mean fluorescence intensity shown, representative of three independent experiments) and (c) p16 using western blot (*n* = 3). (d) CCL2, IL‐6 and IL‐8 were quantified in culture supernatants using cytometric bead array (*n* = 6). Data are represented as mean ± SEM and compared using either a paired *t* test or Wilcoxon matched‐pairs test. *p ≤ 0.05; **p ≤ 0.01; ****p ≤ 0.0001.

Human dermal fibroblasts were isolated from healthy control skin biopsies, senescence was induced using 10 Gy x‐ray ionising radiation. We confirmed the development of senescence 14 days post‐irradiation using an SA‐β‐galactosidase assay (Figure 1a) and showed increased expression of senescence‐associated markers such as cell cycle inhibitors p16 and p21 and DNA‐damage protein γ‐H2AX by western blot and immunofluorescence (Figure 1b,c, Figure S1). Although non‐senescent fibroblasts secrete baseline levels of inflammatory mediators, senescent fibroblasts exhibited an increased production of characteristic SASP mediators including CCL2, IL‐8 and IL‐6 at both Days 7 and 14 post‐irradiation as determined by cytometric bead array (Figure 1d, Figure S2). These mediators were determined as the most significantly increased SASP components in fibroblasts from our previous findings and were therefore used as an indicator for senescence in our assays (Pereira et al., 2019).

**FIGURE 2:**
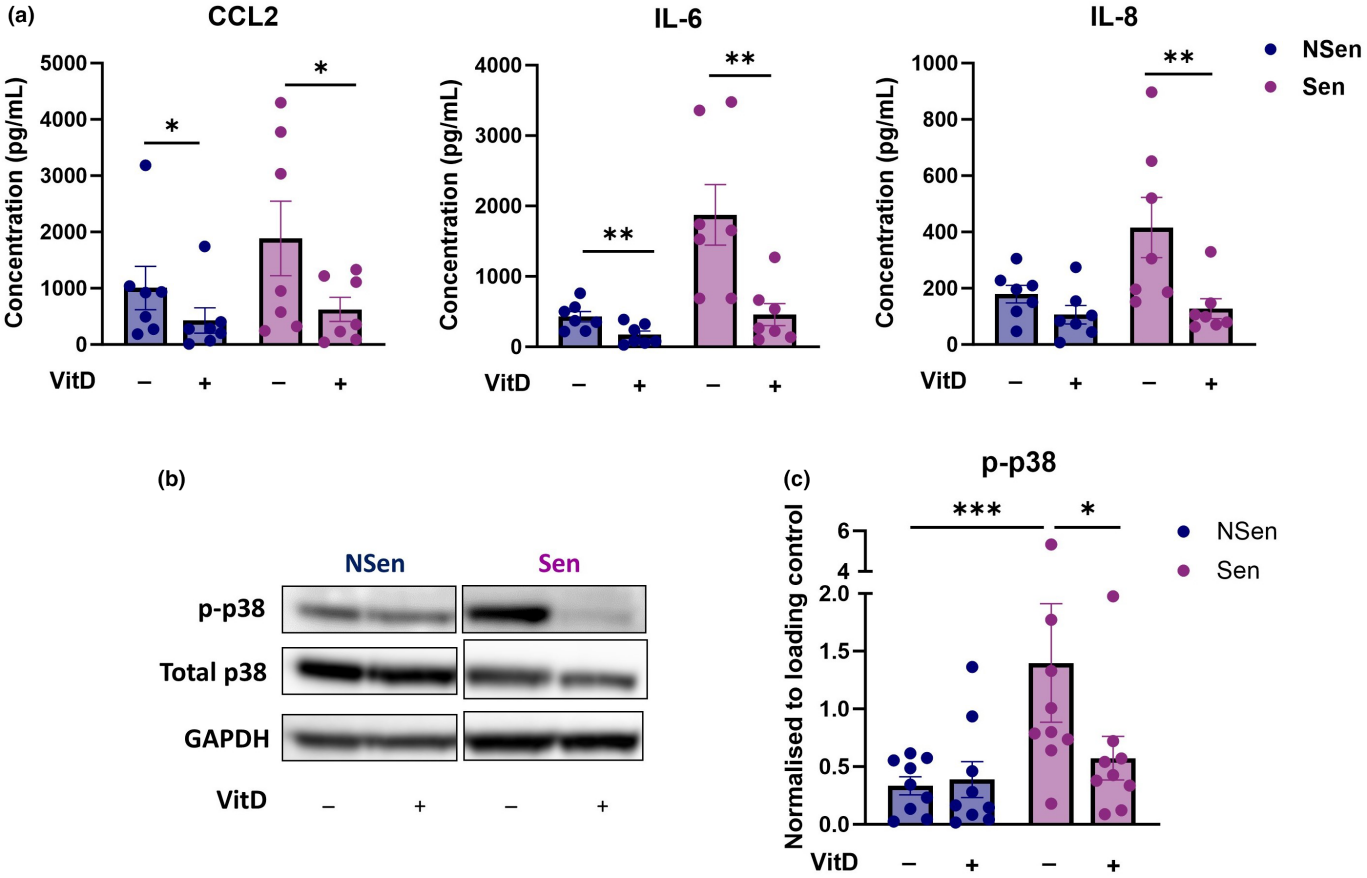
1,25(OH)_2_D_3_ inhibits senescence‐associated mediators in fibroblasts and blocks p38 MAPK. Non‐senescent and senescent fibroblasts were treated with 10 nM 1,25(OH)_2_D_3_ for 7 days. (a) Quantification of CCL2, IL‐6 and IL‐8 was performed using cytometric bead array (*n* = 7). Cell numbers of both non‐senescent and senescent cells were counted after 7 days and the cell number and concentrations in the supernatants of senescent cells were adjusted proportionally to the cell number of their non‐senescent controls. (b) Representative blot of phospho‐p38 MAPK (p‐p38), total p38 MAPK and GAPDH with (c) the cumulative data (*n* = 9) using western blot. Data are represented as mean ± SEM and compared using a one‐way ANOVA with Dunn's multiple comparisons test. *p ≤ 0.05; **p ≤ 0.01; ***p ≤ 0.001.

To test whether vitamin D_3_ regulates the SASP, we first did a dose‐titration of the active form of vitamin D_3_, 1,25(OH)_2_D_3_, using 1 nM, 10 nM and 100 nM and chose the 10 nM concentration as a physiologically relevant dose for all subsequent experiments (Figure S3) (Jeffery et al., 2012). We treated non‐senescent and senescent fibroblasts with 10 nM of 1,25(OH)_2_D_3_ for either 24 h or 7 days. Vitamin D_3_ treatment significantly inhibited CCL2, IL‐8 and IL‐6 by senescent cells on both time points with a much stronger inhibition after a 7‐day treatment (Figure 2a, Figure S4). The relatively low baseline secretion of CCL2 and IL‐6 by non‐senescent cells was also inhibited by this treatment (Figure 2a).

Having previously shown that the SASP is regulated by the p38 MAPK pathway (Freund et al., 2011; Vukmanovic‐Stejic et al., 2018) and that the specific p38 MAPK inhibitor losmapimod could inhibit SASP‐related mediator production in the skin after saline challenge (Chambers, Vukmanovic‐Stejic, Shih, et al., 2021; Vukmanovic‐Stejic et al., 2018), we investigated whether vitamin D_3_ could block p38 MAPK and the SASP in a similar manner. We show that senescent fibroblasts increase their expression of phosphorylated p38 MAPK by western blot analysis and that this was inhibited after vitamin D_3_ treatment (Figure 2b,c). However, this upregulation of phosphorylated p38 MAPK was not seen in vitamin D_3_‐treated non‐senescent cells indicating that the inhibition of p38 MAPK by vitamin D_3_ only occurs in senescent fibroblasts. Furthermore, we show that this effect is sustained for at least 6 days post‐removal of vitamin D_3_ from cell culture media indicating that vitamin D_3_ has possible beneficial long‐term effects on the SASP even after the end of the treatment (Figure S5).

Consistent with our findings, studies have previously shown that upon 1,25(OH)_2_D_3_ treatment of monocytes, the vitamin D receptor (VDR) directly interacts with the promoters of MAPK phosphatase‐1 (MKP‐1) and induces its expression which in turn dephosphorylates p38 MAPK and inhibits its expression (Zhang et al., 2012). Similarly, vitamin D_3_ upregulates MKP‐5 in prostate cells and in keratinocytes to inactivate p38 MAPK (Nonn et al., 2006). Vitamin D_3_ could therefore induce the expression of these phosphatases in senescent fibroblasts as well to indirectly inhibit the p38 MAPK pathway.

However, other pathways are likely to additionally be engaged. To further investigate this, we evaluated p38 MAPK expression on Days 2, 5 and 7 post‐treatment of vitamin D_3_ and show that vitamin D_3_ inhibits p38 MAPK expression only at Day 5 onwards (Figure S6). Furthermore, when fibroblasts are treated in combination with vitamin D_3_ and a specific p38 inhibitor, BIRB‐796, a significant additive effect on the inhibition of p38 MAPK expression and IL‐8 production is observed (Figure S7). This highlights that vitamin D_3_ not only targets the p38 MAPK pathway but is possibly targeting other pathways as well, one of which could be NF‐κB, another regulator of the SASP (Chien et al., 2011). Overall, our findings suggest that one mechanism for the enhanced response to cutaneous VZV challenge in vivo after vitamin D_3_ supplementation was through the inhibition of the SASP produced by senescent stromal cells, that increase in number in the skin during ageing (Pereira et al., 2019).‐.

**FIGURE 3:**
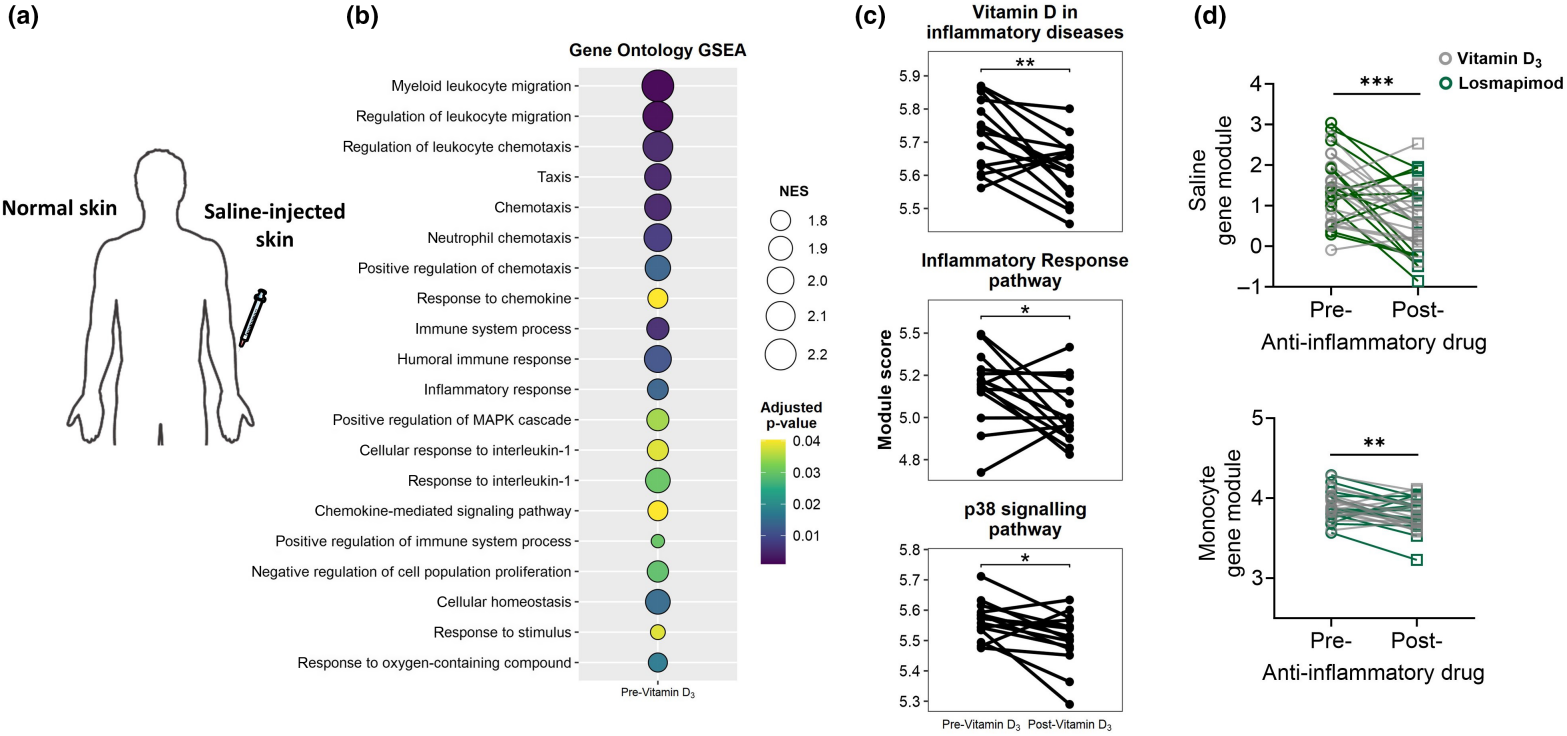
Vitamin D mimics the mechanism of action of losmapimod, a specific p38 inhibitor in vivo. (a) Schematic representation of biopsies collection for RNA‐Seq analysis. Biopsies collected 6 hrs post‐saline injection, before and after anti‐inflammatory drug supplementation. All data were represented as delta change of saline‐injected skin as compared to normal skin. (b) GSEA was performed on saline‐injected versus normal skin biopsies to identify pathways induced by saline injection. Circles represent activated hallmark gene sets (NES > 0) in comparison to normal skin, with the radius proportional to NES. Circle colors represent adjusted *p*‐values for each term. (c) Analysis of previously curated pathways from WikiPathways using ‘Inflammatory response’ (WP453), ‘p38 MAPK signaling’ (WP400) and ‘Vitamin D in inflammatory diseases’ (WP4482) pathways, performed on saline‐injected versus normal skin biopsies before and after vitamin D_3_ supplementation. (d) Saline‐specific module and monocyte‐specific module in saline‐injected skin pre and post either vitamin D_3_ supplementation (grey) or losmapimod treatment (green). Data are compared using a paired *t* test. *p ≤ 0.05; **p ≤ 0.01; ***p ≤ 0.001.

The enhancement of the immune response to VZV in the skin of older individuals after vitamin D_3_ supplementation (Chambers, Vukmanovic‐Stejic, Turner, et al., 2021) was not associated with improved T‐cell function in the periphery or changes in senescent‐like T‐cell subsets in the circulation (data not shown). We concluded that the effect of Vitamin D_3_ on the enhancement of the cutaneous response to VZV challenge was specific to the skin. To investigate this further, we obtained biopsies of unchallenged and saline injected skin from the same older individuals. This was performed both before and after the donors were supplemented for 14 weeks with 6400 IU of 25‐hydroxyvitamin D_3_ (Figure 3a) (Chambers, Vukmanovic‐Stejic, Turner, et al., 2021). We performed RNA‐sequencing (RNA‐Seq) analysis on these biopsies to determine whole gene expression before and after vitamin D_3_ supplementation to identify pathways that were induced by saline injection and how they were modulated by vitamin D_3_ supplementation in vivo. The data were represented as delta change of saline‐injected skin compared to normal skin (Figure 3a). RNA‐Seq analysis identified many inflammation‐related pathways in saline‐injected skin that associated with leukocyte chemotaxis and migration, positive regulation of MAPK and response to IL‐1 (Figure 3b). The increased expression of these pathways were found previously to correlate with decreased responses to VZV challenge in the contralateral arm of the same individuals (Chambers, Vukmanovic‐Stejic, Turner, et al., 2021). We found that vitamin D_3_ supplementation reduced the expression of the genes that are linked to monocyte chemo‐attraction and inflammatory cytokine secretion, for example, CCL8, CXCL‐8, TNF and ICAM‐1 (Chambers, Vukmanovic‐Stejic, Turner, et al., 2021). These inflammatory cytokine‐related genes were no longer differentially expressed after vitamin D_3_ supplementation, and no pathways were found to be upregulated and are therefore not shown in Figure 3b.

We now extend these observations by analysing previously curated pathways from WikiPathways as gene modules (Figure 3c). We show that vitamin D_3_ significantly reduced the scores of Inflammatory response pathway (https://www.wikipathways.org/instance/WP453), p38 MAPK signaling pathway (https://www.wikipathways.org/instance/WP400) and vitamin D in inflammatory diseases pathway (https://www.wikipathways.org/instance/WP4482), the latter being a p38 MAPK phosphorylation pathway which induces the expression of several pro‐inflammatory proteins and was previously found to be downregulated by vitamin D_3_. This prompted us to compare the effects of vitamin D_3_ supplementation to the specific p38 MAPK inhibitor losmapimod, which we used in our previous study (Vukmanovic‐Stejic et al., 2018). We showed that the pre‐treatment of older individuals with this drug, also enhanced cutaneous responses to VZV challenge, like vitamin D_3_ (Chambers, Vukmanovic‐Stejic, Turner, et al., 2021). When pooled together, the two anti‐inflammatory drugs similarly decreased the expression of both a saline‐specific and a monocyte‐specific transcriptional module, generated as previously described (Bell et al., 2016; Chambers, Vukmanovic‐Stejic, Turner, et al., 2021) (Figure 3d). This suggests that vitamin D_3_ and losmapimod both inhibit inflammation and the associated recruitment of monocytes by inhibiting similar signalling cascades that enhance antigen‐specific immune responses during ageing. However, we do not rule out the possibility that vitamin D also inhibits additional inflammatory pathways such as NFkB (Chien et al., 2011).

## 2 CONCLUSION

Senescent dermal fibroblasts are key players in exacerbating inflammation not only through SASP production in the skin but also through their recruitment and activation of inflammatory monocytes. Temporarily inhibiting this inflammatory environment in older people is a key step for enhancing cutaneous antigen‐specific immunity. We showed previously that treatment with anti‐inflammatory drugs like losmapimod in vivo can improve immunity. We now highlight that the use of a readily available natural product, vitamin D_3_, mimics the mechanism of action of losmapimod by inhibiting p38 MAPK to block the SASP. This reduces the recruitment of inflammatory monocytes and overall inflammatory responses in the skin leading to increased immune responsiveness after antigenic challenge in old humans in vivo.

## 3 EXPERIMENTAL PROCEDURE

### 3.1 Isolation and culture of human dermal fibroblasts

Dermal fibroblasts were isolated from 3 mm skin biopsies obtained from the forearm of healthy donors, three females and four males aged between 29 and 72 (Table S1). The study was approved by Ethics Committee of Queen Square (London, United Kingdom) and by the institutional review board (UCL R&D), all donors provided written informed consent prior to the procedure. While we do not take detailed health information from our donors, they do not have inflammatory disease or take immunomodulatory drugs. Briefly, biopsies were digested and cultured using the Whole Skin Dissociation Kit (Miltenyi Biotec). Senescence was induced in primary fibroblasts by exposing them to 10 Gy x‐ray radiation at a dose of 2.4 Gy/min. Cells were then left for 14 days in culture for senescence to develop. Senescence was confirmed using an SA‐βgalactosidase assay (Cell Signaling). All experiments were performed using either early passage non‐senescent fibroblasts from P3 to P5 or senescent fibroblasts.

### 3.2 Immunofluorescence

Non‐senescent and senescent fibroblasts were seeded in a 96‐well Optical bottom plate (4 × 10^3^ cells/well) (Nunc). The following day, cells were fixed with 4% PFA for 10 min permeabilized with 0.1% Triton X100 and then blocked with Dako Protein Block (Agilent). The cells were then incubated with a primary antibody cocktail overnight against p21, γH2AX, Ki67, HLA‐E and MICA/B followed by a secondary antibody cocktail conjugated to AF647 or AF488 for 1 h. Cells were then stained with Phalloidin (Life Technologies) and DAPI for 20 min. The plate was imaged using the CellDiscoverer 7 microscope (Zeiss) and images were analysed using the Zeiss Zen Software.

### 3.3 Treatment with vitamin D_3_ or BIRB‐796

After senescence was established, non‐senescent and senescent fibroblasts were seeded into either a 48‐well plate (at 2 × 10^4^ cells/well) for cytokine quantification or a 6‐well plate (at 15 × 10^4^ cells/well) for western blotting. The following day, cells were treated with either vehicle or 1,25(OH)_2_D_3_ (Enzo Life Sciences) at 10 nM and/or BIRB‐796 (Selleckchem) at 5 μM for either 2, 5 or 7 days with media replacement every 2–3 days. The medium was then replaced for the last 24 h to collect culture supernatants. A dose–response curve was performed using either 1, 10 or 100 nM of 1,25(OH)_2_D_3_ for 24 h. To evaluate longer term effect of vitamin D_3_, cells were treated with 1,25(OH)_2_D_3_ at 10 nM for 7 days and then 1,25(OH)_2_D_3_ was replaced with fresh media and harvested either on the same day or on day 2 and 6 post‐removal of 1,25(OH)_2_D_3_.

### 3.4 Cytometric bead array

To assess the SASP in culture supernatants, concentrations of IL‐6, IL‐8 and CCL2 were assessed using cytometric bead array (BD Biosciences). Data were acquired using a BD Verse Flow Cytometer (BD Biosciences) and analysed using FCAP array v3 Software (BD Biosciences). Concentrations of cytokines were normalized to cell numbers counted at the time of supernatant collection.

### 3.5 Western blot

Cell pellets were lysed with RIPA buffer (Sigma‐Aldrich) supplemented with phosphatase and protease inhibitor (Cell Signalling) for 30 min on ice. Protein concentrations were determined using BCA protein assay (ThermoScientific). 25–30 μg of proteins were then diluted using the SDS sample buffer containing reducing agents (Life Technologies) and denatured at 95°C for 5 min. Extracts were separated by protein electrophoresis using a 10% Bis‐Tris pre‐cast gel (Life Technologies) and then transferred onto a Hybond PVDF membrane (GE Healthcare). The membranes were then blocked in ECL blocking agent and then probed overnight with antibodies against phospho‐p38 MAPK, p38 MAPK, p16 INK4A, GAPDH and β‐actin followed by HRP‐conjugated secondary antibodies (Cell Signalling). Antibodies were detected using the ECL detection kit (GE Healthcare) and bands were visualized using the Cytiva Amersham ImageQuant 800 imaging system. Before re‐probing, membranes were stripped using Restore Stripping Buffer (ThermoScientific). All images were analysed using ImageJ Software. Protein expression was normalized to expression levels of GAPDH in each corresponding lane.

### 3.6 RNA‐Seq analysis

Biopsies of either normal skin or saline‐injected skin were obtained at 6 h from 18 healthy older individuals, before and after a 14‐week 6400 IU vitamin D_3_ supplementation for RNA‐Seq analysis as previously described (Chambers, Vukmanovic‐Stejic, Turner, et al., 2021) or from 18 healthy older individuals, before and after a 4‐day losmapimod (GW856553) treatment of 15 mg twice daily for 4 days as previously described (Vukmanovic‐Stejic et al., 2018). To investigate the impact of vitamin D_3_ supplementation, paired‐end raw RNA‐Seq data were subject to quality control using FastQC and Trimmomatic for trimming low quality bases and adapters removal. Afterwards, samples were aligned to the human genome (Ensembl Release 101) using Salmon with the ‐‐gcBias and –validateMappings flags and transcript abundance was assembled using tximport package in R. Counts were normalized using TMM method in EdgeR package. The sva package was used to infer surrogate variables (SVs) to catch unwanted variation in the dataset. Batch and SVs effects were removed from the normalized matrix for subsequent analysis except for differential testing. Before differential testing, the data were inspected for outliers using the Robust PCA method (Chen et al., 2020). For differential expression, the limma‐voom approach from the limma package was used while blocking for subject. In addition, both batch and SVs that did not correlate with the groups of interest were included in the design model to account for unwanted effects. Genes that had adjusted *p*‐values lower than 0.05 and log_2_ fold‐changes greater than 1 (parameter fc = 1.2 in treat() function from limma) were considered as differentially expressed genes (DEGs). DEGs were further subject to Gene Set Enrichment Analysis using the clusterProfiler package to investigate Gene Ontology Biological Processes. After obtaining the list of enriched processes, the list was filtered for redundancy of terms using the simplify() function with a cut‐off of 0.75 and selecting by maximum enrichment score. Module scores from gene modules obtained from WikiPathways were calculated as the average log expression of all genes in each module, and statistical differences were calculated using Wilcoxon signed‐rank tests for paired data. All plots were made using ggplot2 package. To compare the effects of vitamin D_3_ and losmapimod, datasets from both treatment courses were pooled together and assessed for saline‐specific and monocyte‐specific transcriptional modules generated as previously described (Chambers, Vukmanovic‐Stejic, Turner, et al., 2021; Nonn et al., 2006).

### 3.7 Statistical analysis

All results are shown as mean ± SEM. Data were assessed for normality and different groups were compared using either a paired Student's *t* test for two groups or one‐way ANOVA for more than two groups. All data were analysed using Graphpad Prism v8 Software. *p* values ≤ 0.05 were considered as statistically significant.

## AUTHOR CONTRIBUTIONS

SS designed and performed the experiments and wrote the manuscript, CHF performed the bioinformatics analysis, PL performed experiments, PS performed clinical support and sample collection, MHAR was involved in the overall design of the project, DOG provided intellectual guidance for the direction of the project, ANA obtained the fundings, designed the experiments, coordinated the collaborative interactions between research groups and wrote the manuscript, ESC designed the experiments, provided early preliminary data and contributed to writing and editing the manuscript.

## CONFLICT OF INTEREST STATEMENT

The authors declare no conflict of interest.

## Supporting information


Table S1.
Figures S1–S7.

## Data Availability

RNA‐Seq data relating to the vitamin D_3_ replacement study that support the findings of this work have been deposited in NCBI Gene Expression Omnibus under accession GSE156212. RNA‐Seq data relating to the losmapimod study that support the findings of this work have been deposited in the NCBI Gene Expression Omnibus under accession GSE130633.
