# Supplementary material for: Vitamin D3 inhibits p38 MAPK and senescence‐associated inflammatory mediator secretion by senescent fibroblasts that impacts immune responses during ageing
Source: Aging Cell. 2024 Jan 29;23(4):e14093. doi: 10.1111/acel.14093 (PMC11019144; doi:10.1111/acel.14093)

Supplementary Table 1.

Age (years old)	Gender
29	F
29	M
33	F
45	M
52	F
63	M
72	M

Supplementary Figure 1.

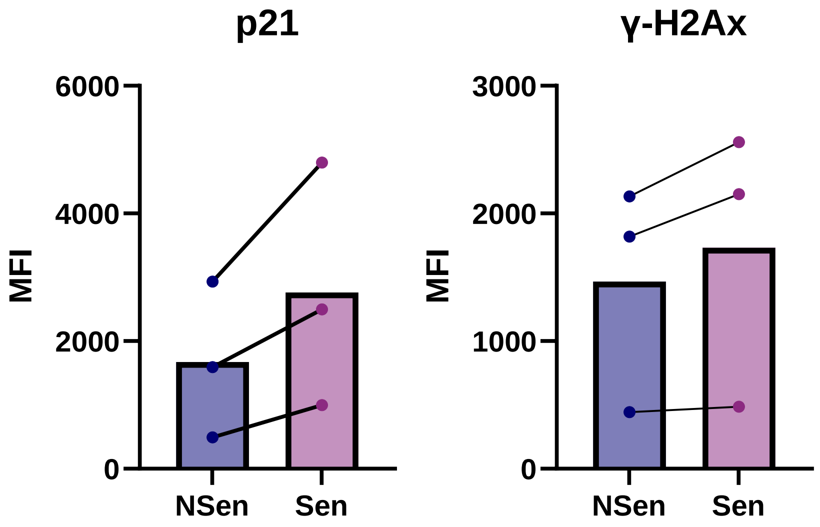

Supplementary Figure 2.

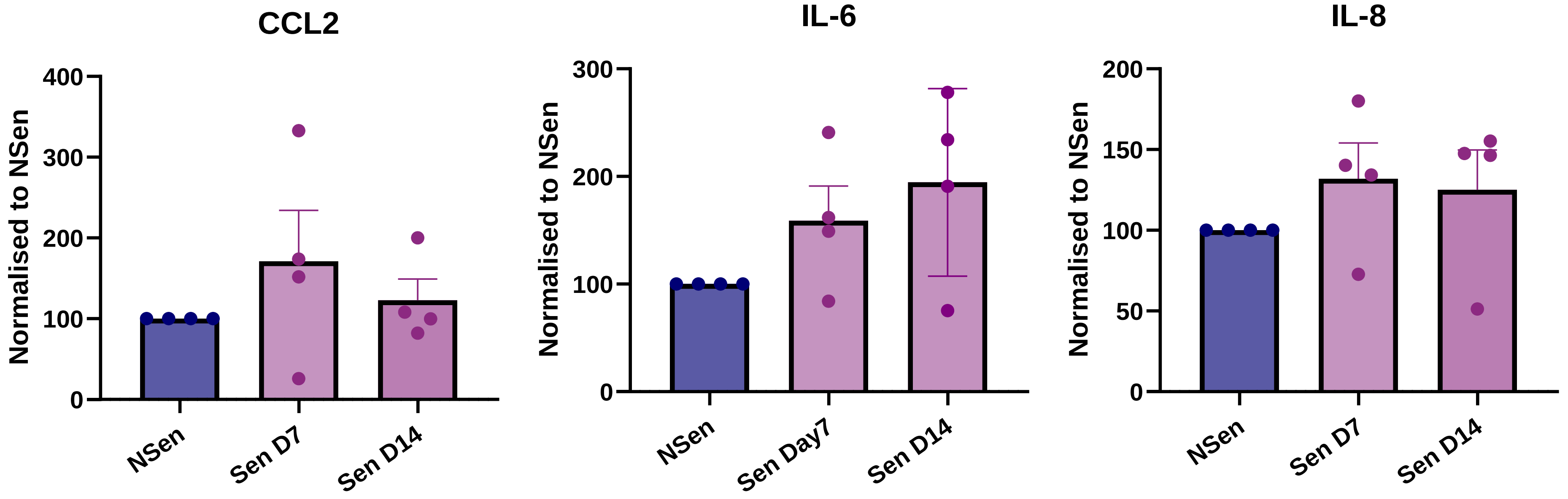

Supplementary Figure 3.

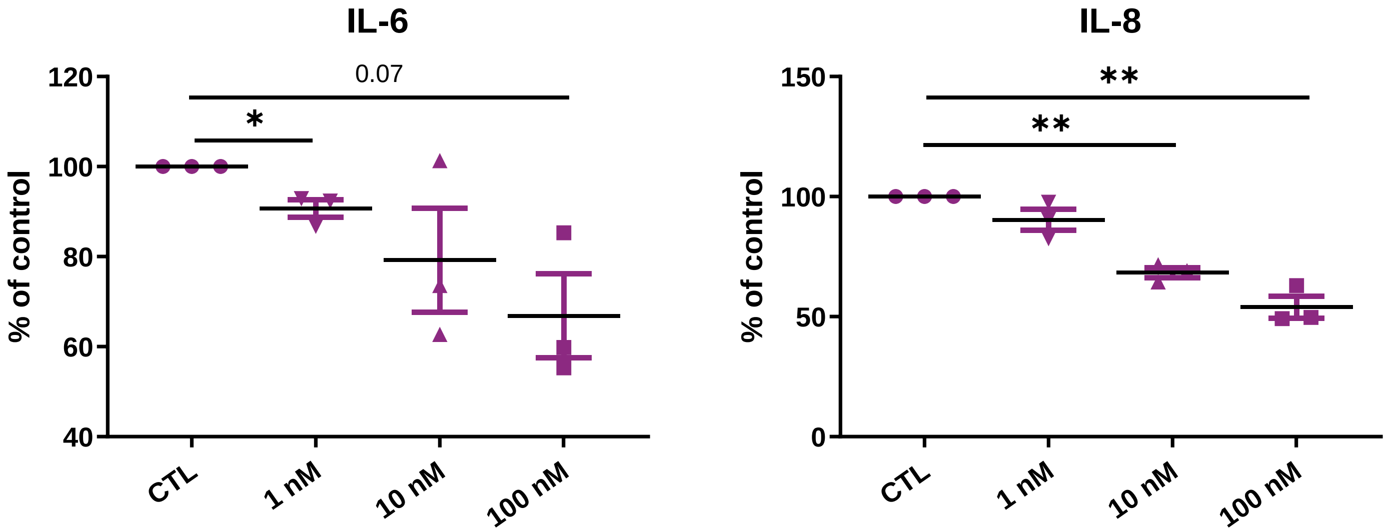

Supplementary Figure 4.

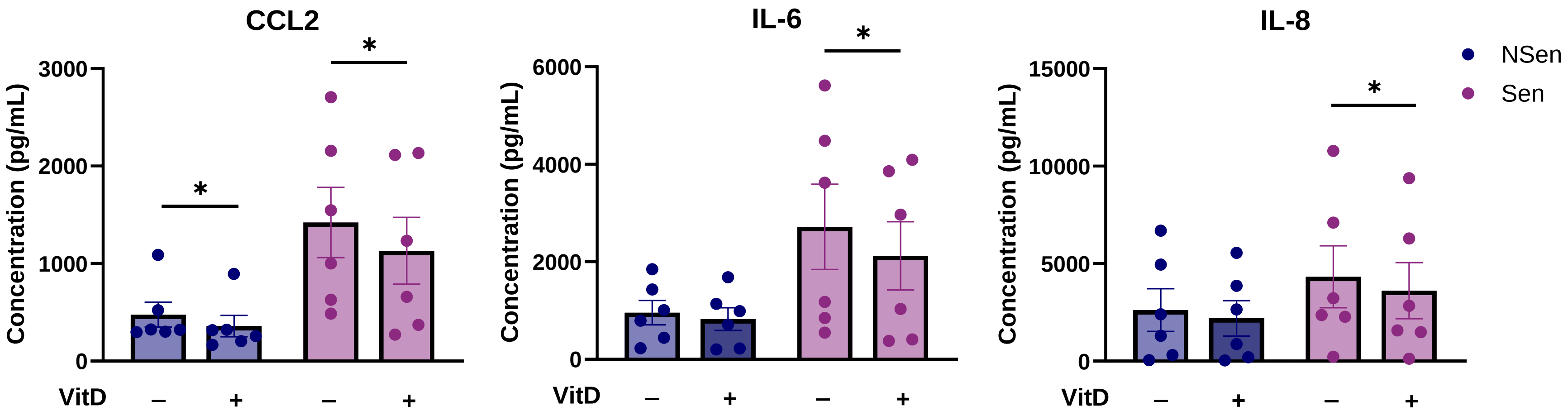

Supplementary Figure 5.

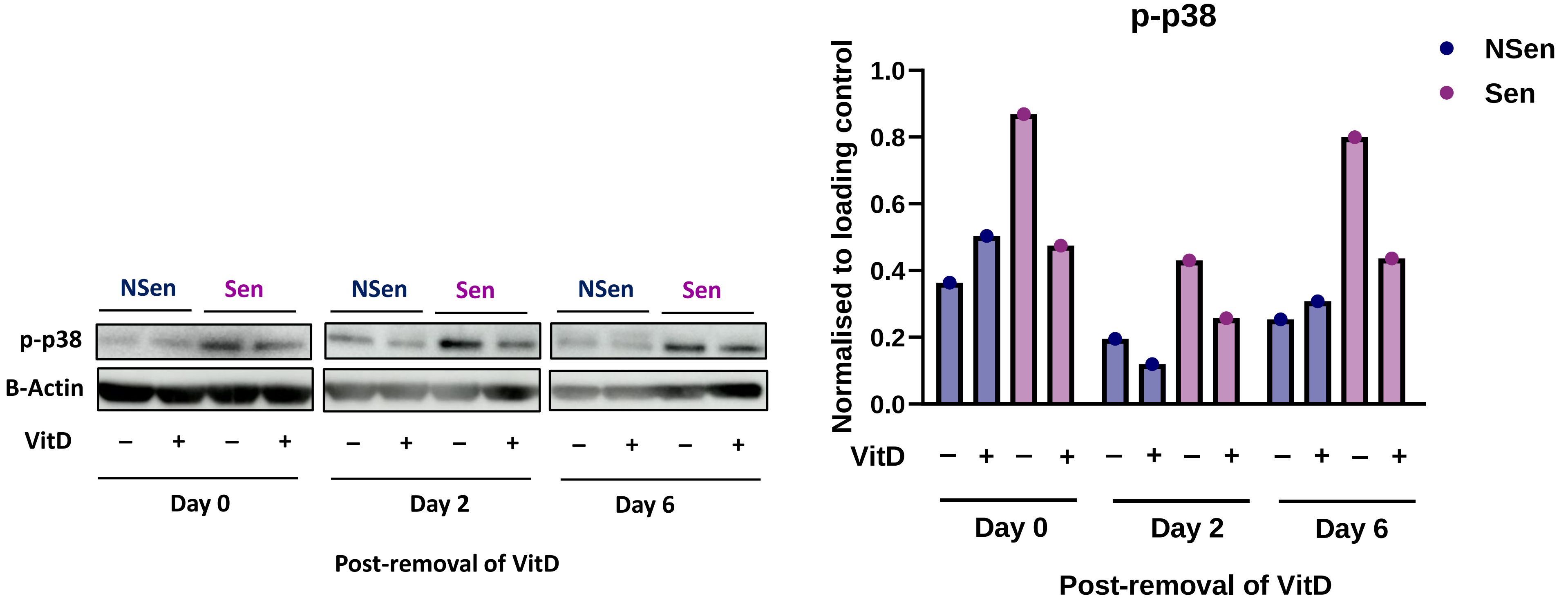

Supplementary Figure 6.

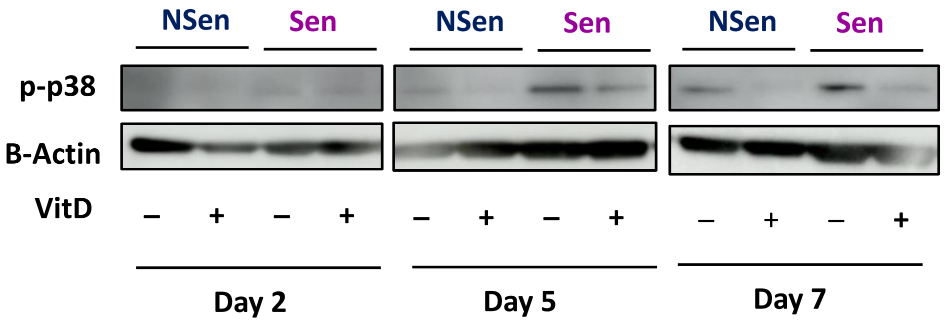

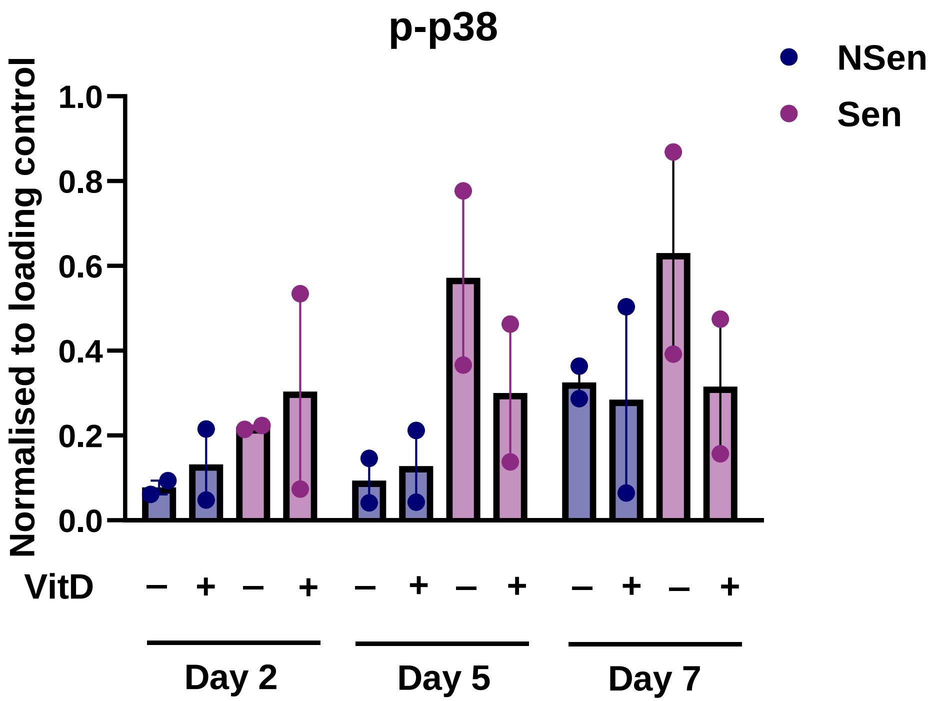

Supplementary Figure 7.

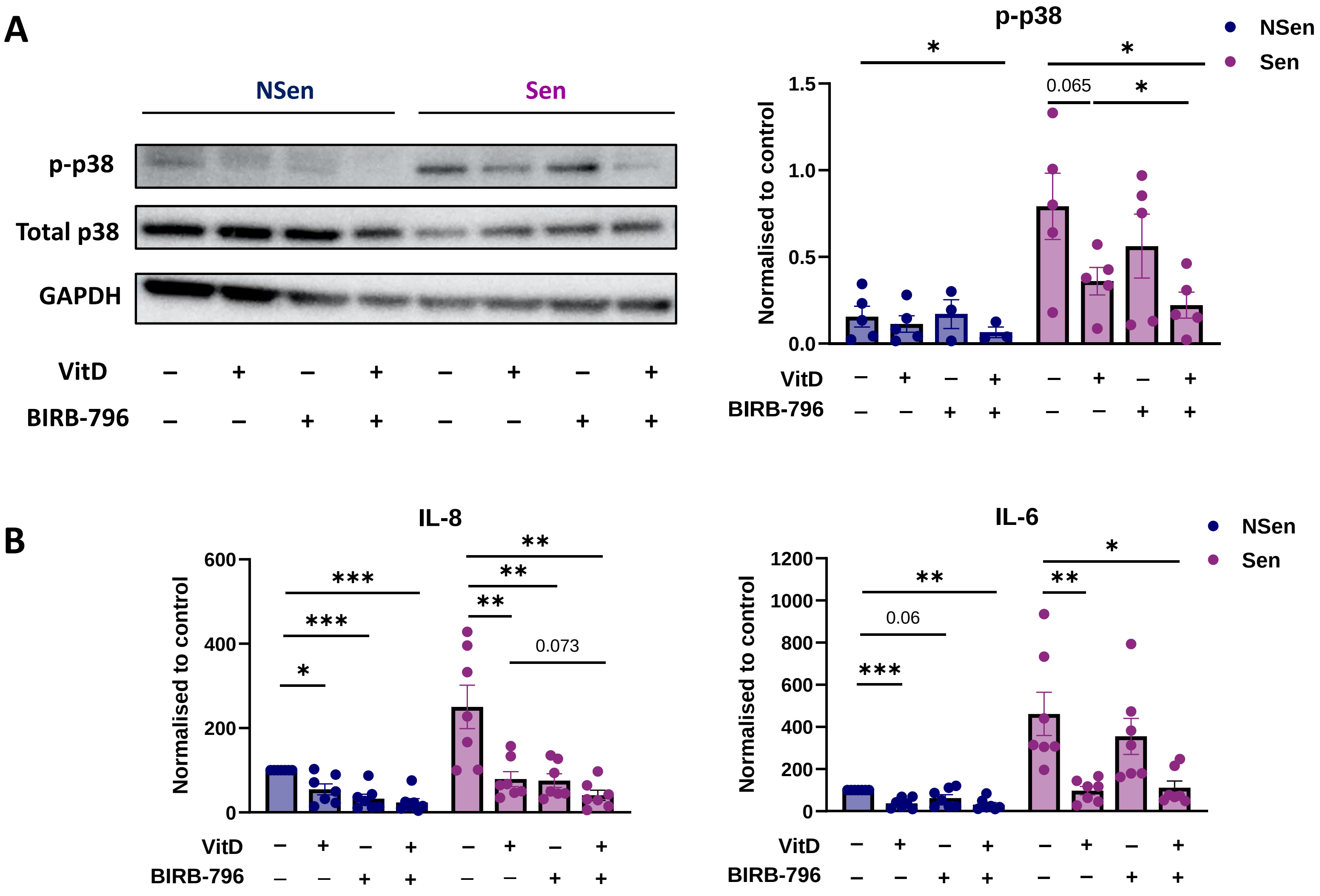

Supplement: Supplementary file 1 — Table S1. Figures S1–S7. [file ACEL-23-e14093-s001.pdf]
